# MicroRNA regulation of the proliferation and apoptosis of Leydig cells in diabetes

**DOI:** 10.1186/s10020-021-00370-8

**Published:** 2021-09-08

**Authors:** Li Hu, Shaochai Wei, Yuqi Wu, Shulin Li, Pei Zhu, Xiangwei Wang

**Affiliations:** 1 Shenzhen University South China Hospital, Shenzhen University, Shenzhen, 518111 People’s Republic of China; 2 Department of Urology & Carson International Cancer Center, Shenzhen University General Hospital & Shenzhen University Clinical Medical Academy Center, Shenzhen University, NO.1098, Xueyuan Road, Shenzhen University City, Nanshan District, Shenzhen, 518055 People’s Republic of China; 3 Department of Physiology, Shantou University of Medical College, Shantou, 515041 People’s Republic of China

**Keywords:** Testicular damage, Diabetes mellitus, Testosterone, Small RNA, MAPK signalling pathway

## Abstract

**Background:**

The number of patients with diabetes is increasing worldwide. Diabetic testicular damage can cause spermiogenesis disorders and sexual dysfunction. We thus explored the role of miRNAs in diabetic testicular damage, and revealed that they could serve as effective prevention and treatment therapeutic targets.

**Methods:**

Streptozotocin (STZ) was used to generate a rat model of type 2 diabetes. Rat testicular tissues were used for miRNA and mRNA sequencing. Through bioinformatics analysis, we constructed an miRNA–mRNA diabetic testicular damage regulatory network and screened for key miRNAs. We also used Leydig cells to generate a diabetic cell model and detected the downstream target genes of miRNAs, secretion of testosterone, and proliferation and apoptotic levels to elucidate the role and mechanism of the selected miRNAs in diabetic testicular damage.

**Results:**

Using second-generation sequencing, we identified 19 differentially expressed miRNAs and 555 mRNAs in the testes of diabetic rats. Based on computational prediction of targets and negative regulation relationships, we constructed a miRNA–mRNA regulatory network, including 12 miRNAs and 215 mRNAs. KEGG enrichment analysis revealed that genes were more concentrated on the survival signalling pathway. Based on this, we screened 2 key miRNAs, miR-504 and miR-935. In vitro, glucose could induce an increase in miR-504 and miR-935, whereas a decrease in MEK5 and MEF2C in a dose-dependent manner. Overexpression of miR-504 and miR-935 led to the decreased expression of MEK5 and MEF2C, decreased proliferation rate of Leydig cells, increased apoptotic rate, and decreased secretion of testosterone. Whereas, knockdown of miR-504 and miR-935 displayed opposite tendencies.

**Conclusions:**

miRNAs play important roles in diabetic testicular damage. miR-504 and miR-935 might regulate testicular damage through the classic survival pathway of MEK5-ERK5-MEF2C. Targeted inhibition of miR-504 and miR-935 could reverse the high-glucose-induced testicular complications, thus posing as a potential therapeutic approach in diabetic testicular injury.

**Supplementary Information:**

The online version contains supplementary material available at 10.1186/s10020-021-00370-8.

## Introduction

According to the data of the International Diabetes Federation (IDF), changes in the lifestyle of people and acceleration in the aging process of the population, which have been associated with the worldwide economic development, have prompted an increasing number of people affected with diabetes annually. More specifically, diabetes mellitus has been reported to affect an estimated 463 million people globally (Saeedi 2019). To date, medical research has mainly focused on a deeper understanding of diabetes-induced complications, such as diabetic retinopathy, cardiovascular diseases, kidney diseases, and peripheral neuropathy (Cole and Florez 2020). As the incidence of diabetes is increasing annually, people have now started to pay increasing attention to the diabetes-inflicted damages in the reproductive system (Maresch et al. 2018), in addition to the commonly known damage to the cardiovascular and kidney systems. Apart from the tissue structure of male reproductive organs and the changes in the proliferation and function of germ cells, increasing attention has also been paid to issues, such as the synthesis of reproductive hormones and secretion disorders, sexual dysfunction, and reproductive ability. Testosterone is known to be mainly secreted by the testes. According to epidemiological statistics, diabetes has been shown to affect the sperm quality and fertility of patients (Kautzky-Willer et al. 2016). Approximately 90% of male patients with diabetes mellitus have varying degrees of testicular dysfunction. In addition, male diabetic patients are characterized by hypotestosteronemia, which causes decreased spermatogenesis. Restriction in the growth of sex organs and development of secondary sex characteristics has been reported to lead to diabetic erectile dysfunction. The risk of sexual and reproductive dysfunction is 5–10 times higher in patients with diabetes than in non-diabetic individuals (Shi et al. 2017; Tavares et al. 2019). Furthermore, diabetes is known to seriously affect the physical and mental condition of patients (Taieb et al. 2019). Diabetes-induced reproductive dysfunction is known to be mainly caused by testicular tissue damage; however, the precise molecular mechanism is not yet clearly understood. To date, no specific therapeutic agents are available for its treatment.

MicroRNAs (miRNAs) have been reported to be involved in the organ damages induced by various types of diabetes. For this reason, miRNAs have been suggested to be critical therapeutic targets for the treatment of diabetic testicular damage (Regazzi 2018; Zhang et al. 2017). However, testicular damage has been rarely reported in diabetic rat models. Here, we used RNA sequencing (RNA-seq) to identify the miRNA–mRNA regulatory network in the diabetic testicular tissues by looking for miRNAs that play key roles in diabetic testicular damage. We also performed a preliminary functional study.

## Materials and methods

### Animal models

All animal experiments were performed at the Lab Animal Center of Shantou University Medical College and were approved by The Medical Animal Care & Welfare Committee of Shantou University Medical College (SUMC2019-407). All rat strains were purchased from the Animal Research Center of Shantou University Medical College. To induce experimental Type 2 diabetes mellitus (T2DM), Sprague–Dawley (SD) rats weighing 250–300 g were actuated by sustaining a high-fat diet feeding routine for 1 mo until their weight reached 300–400 g. Subsequently, diabetes was induced by a single intraperitoneal injection of 35 mg/kg streptozotocin (STZ) (Sigma Aldrich, Shanghai, China; mixed in freshly prepared cold 0.1 mol/L citrate cradle, pH 4.2–4.5). Control rats received an intraperitoneal injection of citrate buffer. The levels of blood glucose were determined in tail vein blood samples using the OneTouch Ultra system (Johnson and Johnson Medical, Shanghai, China) 3 d after the STZ infusion, and checked weekly. Only rats with a constant blood glucose concentration higher than 16.7 mmol/L were considered diabetic. (Qiao et al. 2018; Skovsø 2014).

### RNA sequencing

Eight weeks after the STZ injection, total RNA was extracted from the testes of animals in each group using the TRIzol reagent (Invitrogen, Carlsbad, CA, USA). All RNA samples were quantitatively and qualitatively checked through 1% agarose electrophoresis and Nanodrop measurement before sequencing. In the case of mRNA sequencing, the paired-end sequencing mode of the Illumina Hiseq 3000 (Illumina, San Diego, CA, USA) sequencing platform was used for high-throughput sequencing, while the FastQC software (available online at http://www.bioinformatics.babraham.ac.uk/projects/fastqc) was used for quality control analysis of the preprocessed data. The BWA software (https://sourceforge.net/projects/bio-bwa/files/) was used to compare the pre-processed data to the rRNA sequence database. The STAR software (available at http://gingeraslab.cshl.edu/STAR) was used to compare the pre-processed sequence with the reference genome sequence of the sequenced species and to use the RSEQC comparison file for statistics.

The String Tie software (http://ccb.jhu.edu/software/stringtie/) was used to assemble the transcripts in samples based on the position information file of the known transcripts in the genome as a guide. Fragments per kilobase million (FPKM) was used to calculate each transcript expression level, while the DESeq2 software (http://www.bioconductor.org/packages/release/bioc/html/DESeq2.html) was used to screen differential transcripts. The single-ended 50 bp sequencing mode of the Illumina Hiseq3000 sequencing platform was used to perform miRNA high-throughput sequencing of samples The primer and adaptor sequences were removed from the original data and reliable sequencing fragments were selected. The types and quantities of small RNAs were then counted. According to the family classification criteria of the miRBase database, known miRNAs were family annotated, and new miRNAs were family analysed according to the seed sequence. The calculation of miRNA expression used counts per million (CPM) to calculate the metric, while the DESeq software was used to screen differentially expressed miRNAs. The obtained RNA-seq data were deposited in the GEO database (Accession code: GSE169131).

### Histological assessment

Testicular tissues were fixed in animal testicular tissue fixative (Servicebio, Wuhan, China) for 24 h and then transferred to 70% ethanol for storage. After embedding of tissues in paraffin, 5-μm thick sections were obtained. Tissue morphology was observed using hematoxylin and eosin (HE) staining according to the manufacturer’s instructions (Solarbio, Beijing, China).

### TUNEL assay

Paraffin-embedded testicular tissue sections were used for the TUNEL assay to determine apoptotic cells in tissues. TUNEL-positive cells were detected using a DNA Fragmentation Detection Kit (Merck Millipore, Billerica, MA, USA), according to the recommended protocol.

### Cell culture, transfection, and reagents

R2C cells purchased from the China Infrastructure of Cell Line Resources (Beijing, China) were transfected with miRNA mimics for gain-of-function experiments, and miRNA inhibitors (GenePharma, Shanghai, China) for loss-of-function experiments. Cell transfection was performed using Lipofectamine 3000 (Invitrogen, Carlsbad, CA, USA) following the manufacturer’s instructions. miR504 mimic (sense:5′-AGACCCUGGUCUGCACUCUGUC-3′, antisense: 5′-CAGAGUGCAGACCAGGGUCUUU-3′), mi504 inhibitor (5′-GACAGAGUGCAGACCAGGGUCU-3′), miR935 mimic (sense:5′-CCAGUUACCGCUUCCGCUACCGC-3′, antisense: 5′-GGUAGCGGAAGCGGUAACUGGUU-3′), mi935 inhibitor (5′-GCGGUAGCGGAAGCGGUAACUGG-3′), mimicNC (sense:5′-UUCUCCGAACGUGUCACGUTT-3′, antisense: 5′-ACGUGACACGUUCGGAGAATT-3′) and inhibitor NC(5′-CAGUACUUUUGUGUAGUACAA-3′) were transfected at a final concentration of 50 nM for 24 h. Cell culture was maintained in DMEM (GIBCO, Grand Island, NY, USA) supplemented with 10% FBS (GIBCO,) in a humidified air incubator with 5% CO_2_ at 37 °C. Leydig cells were exposed to normal (5 mM) or moderately high (15 mM) or high (30 mM) glucose concentrations for 48 h according to the previous study (Karpova et al. 2020).

### Real-time quantitative PCR (RT-qPCR)

Blood samples were obtained from patients with diabetes and healthy donors at Shenzhen University General Hospital. This project was approved by the ethics committee of the Shenzhen University. Total RNA was extracted from blood using a QIAamp RNA Blood Mini Kit (QIAGEN, Duesseldorf, Germany). Total RNA from tissues and cells was extracted using a TaKaRa MiniBEST Universal RNA Extraction Kit (TaKaRa, Tokyo, Japan) following the manufacturer’s instructions. For the quantification of miRNA by qPCR, reverse transcription and RT-qPCR were performed using the Mir-X miRNA RT-qPCR TB Green® Kit (TaKaRa) and normalized to U6. The entire sequence of mature miRNA was used as miRNA specific, 5′ primer (miR-504, 5′-AGACCCUGGUCUGCACUCUGUC-3’ miR-935, 5′-CCAGUUACCGCUUCCGCUACCGC-3′; miR-484, 5′-UCAGGCUCAGUCCCCUCCCGAU-3′; miR-301a-5p, 5′-GCUCUGACUUUAUUGCACUAC-3′; U6, 5′-CGTTCACGAATTTGCGTGTCAT-3′). The 3′ primer used in the qPCR was the mRQ 3′ primer supplied with the kit. Reverse transcription of mRNA was performed using the PrimeScript™ RT Master Mix (TaKaRa), while RT-qPCR was performed using the One Step TB Green® PrimeScript™ RT-qPCR Kit II (TaKaRa) and normalized to β-actin. The primers used were as follows: MEK5 forward primer 5′-TCGTGCCATGGAGAACCA-3′, reverse primer 5′-CGCGCCACTATTTGGAATCT-3′; MEF2C forward primer 5′-ACCACCACCCCATCGAGATA-3′, reverse primer 5′-GGAGTGGAATTCGTTCCGGT-3′; β-actin forward primer 5′-ATGGATGACGATATCGCTGC-3′, reverse primer 5′-CTTCTGACCCATACCCACCA-3′. The 2‐ΔΔCq method was employed to compare the relative levels of expression of miRNA and mRNA (Livak and Schmittgen 2001).

### Western blot analysis

Western blot analysis was performed according to previously published methods. R2C cells were washed once with cold PBS (GIBCO) and lysed in RIPA buffer (Sigma-Aldrich, St. Louis, MO, USA) containing protease inhibitors. Total protein was separated by 10% SDS–PAGE, followed by transfer to polyvinylidene difluoride membranes (Millipore Corp, Billerica, MA, USA). Membranes were blocked with 5% skim milk at 25 to 30 °C for 1 h. Membranes were then incubated with primary rabbit anti-rat antibodies against MEF2C (1:1000; Abcam, Cambridge, MA, USA), MEK5 (1:1000; Abcam Cambridge, MA, USA), and β-actin (1:5000; Cell Signaling Technology, Danvers, MA, USA) overnight. Membranes were then washed thrice with TBST(Millipore Corp, Billerica, MA, USA), followed by incubation with anti-rabbit IgG horseradish peroxidase secondary antibody (1:5000; Cell Signaling Technology) for 1 h at 25 °C. Finally, immunoreactive bands were visualized using the ECL reagent (Sigma-Aldrich). Relative levels of protein expression were quantified using the Image J software (NIH Image J 2.0v system, Bethesda, MD, USA) and normalized to β-actin.

### Testosterone enzyme linked immunosorbent assay (ELISA)

Total testosterone was measured using the Rat or Human Testosterone ELISA kit (Cusabio, Wuhan, China) according to the manufacturer’s instructions. After testis tissue was added to HEPES in proportion, the tissue was grinding, and the supernatant was taken for ELISA. Meanwhile, the serum was used in direct assays. A standard curve was constructed using GraphPad Prism (GraphPad Prism c8.0, GraphPad Software, San Diego, CA, USA), applying a sigmoidal 4-parameter logistic fit. The concentration of testosterone (ng/mL) was determined based on this curve.

### CCK8 analysis for cell viability

Cell viability was measured using a Cell Counting Kit-8 (Dojindo, Kumamoto, Japan) according to the manufacturer’s instructions. Briefly, 1 × 10^4^ R2C cells were seeded in 96-well plates with 30 mM high-glucose DMEM after transfection with respective oligos (miRNA mimics and inhibitors). CCK-8 solution (10 μL) was added to each well for 1 h and the optical density was measured at 450 nm using a microplate reader (Beckman Coulter, Miami, FL, USA) for estimation of viable cells. Samples in each group were tested every 24 h for 5 days and the proliferation curves were plotted.

### Apoptosis analysis

Apoptosis was measured using the Annexin V-FITC Apoptosis Detection Kit (Dojindo) according to the manufacturer’s protocol. R2C cells were harvested by centrifugation, mixed, washed twice with PBS, and re-suspended in binding buffer at a final density of 10^6^ cells/mL. Annexin V-FITC (5 μL) was added to 100 μL of the cell suspension, followed by the addition of 5 µL PI solution. The cell suspension was mixed and incubated for 15 min at 25 °C in the dark. Subsequently, 200 μL of binding buffer was added, and cells were analyzed by flow cytometry using CytoFLEX (Beckman Coulter, Miami, FL, USA). Data were analyzed using the Flowjo software (Flowjo 10.4v, Ashland, OR, USA).

### Statistics

Statistical analysis was performed with GraphPad Prism version c8.00. Quantitative data are reported as mean ± SD and binary data by counts. Significance between 2 groups was determined by Mann–Whitney U as appropriate. For comparison between multiple groups, Kruskal–Wallis test was used. A p-value < 0.05 was considered significant.

## Results

### Diabetes led to testicular damage and decreased androgens

**Fig. 1:**
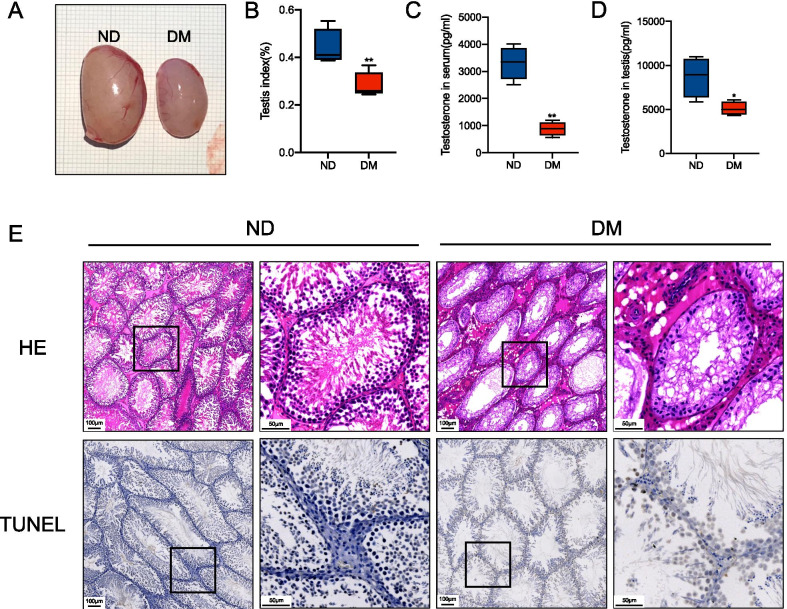
Effects of diabetes on testicular function and apoptosis. Eight weeks after diabetes was established, the right testis of each rat was removed and separately photographed (**A**) and the testis index {(testis weight/body weight) × 100%} was calculated (**B**). Concentrations of serum (**C**) and testicular (**D**) testosterone detected by ELISA in each group. Representative hematoxylin & eosin (H&E) and TUNEL staining of rat testicular tissues from ND (first 2 panels) and DM (last 2 panels) groups. For a better comparison, the second panel in each group is a partially enlarged panel (black box) of the first panel. Scale bar = 100 μm (first panel) and 40 μm (second panel) (**E**). Data are presented as mean ± SD.*p < 0.05 **p < 0.01 compared with the ND group

We generated the DM model in adult male Sprague Dawley rats. We observed that at 8 week after the STZ injection, the DM rats showed a significant decrease in the testicular index (testis weight/body weight × 100%) when compared with the control (Fig. 1A and B). We also found that the serum and testicular tissue levels of testosterone were decreased in DM rats (Fig. 1C and D). Histological analyses revealed that, in contrast to controls, all DM testes displayed a striking reduction of spermatogenesis in the seminiferous tubules. Meanwhile, we observed an apparent increase in the number of apoptotic sperm cells and somatic cells, especially in Leydig cells, as revealed by the TUNEL assay (Fig. 1E). Thus, these results reproduced previous findings and confirmed that diabetes causes testicular cell injury and apoptosis, decreasing androgens and spermatogenesis (Cheng et al. 2020; Khosravi et al. 2019). Based on this, we concluded that diabetes destroys the physiological structure of normal testes in rats.

### miRNA–mRNA integrated profiling of testis in diabetic rats

**Fig. 2:**
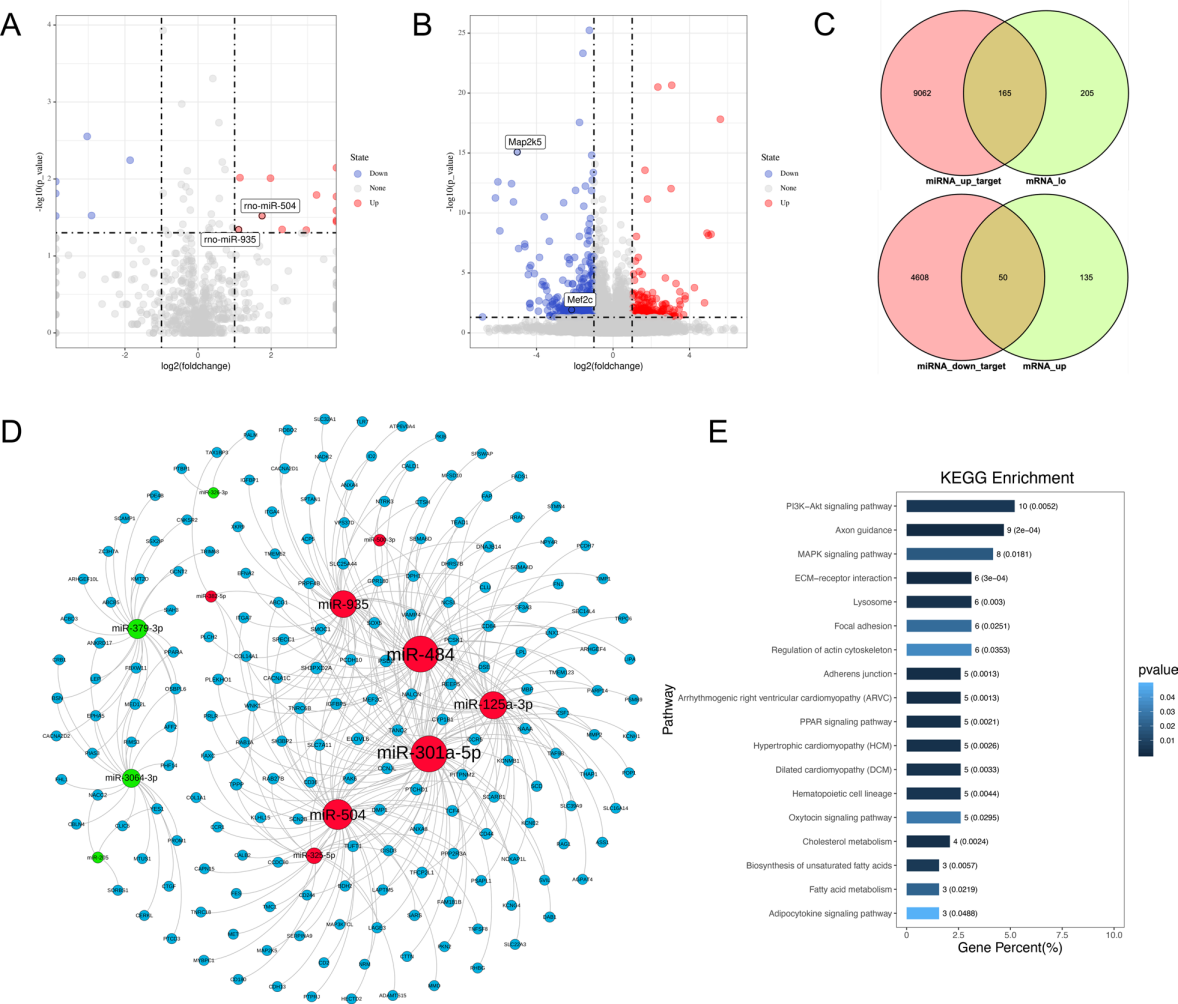
Bioinformatics analysis of testicular miRNA by RNA sequencing. Volcano plot analysis of differentially-expressed miRNAs (**A**) and mRNAs (**B**) in the diabetic vs. normal testis from ND and DM rats. The log2 transformation of the fold change in the expression of miRNAs and mRNAs between diabetic and normal testes from each group is plotted on the x-axis. The log p-value (base 10) is placed on the y-axis. Differentially-expressed miRNAs and mRNAs (fold change ≥ 1) are indicated in red (upregulated miRNAs and mRNA in diabetic testis) and green (downregulated miRNAs and mRNA in diabetic testis). Upregulated (miRNA_up_target) and downregulated (miRNA_down_target) miRNA-target genes were predicted online using TargetScan (http://www.targetscan.org/). The overlapping target genes and downregulated (mRNA_lo) or upregulated (mRNA_up, **C**) mRNAs were identified via Venn diagrams. The miRNA–mRNA regulation networks were constructed using the Gephi software (**D**). Red dots represent upregulated miRNAs, whereas green dots indicate downregulated miRNAs, and blue dots indicate downstream target genes. KEGG analysis of upregulated and downregulated mRNAs in the miRNA–mRNA regulation networks (**E**)

We extracted the total RNA from diabetic and non-diabetic testes and processed them for small RNA-Seq and RNA-Seq, as previously described. Bioinformatics analysis demonstrated the differential expression of 19 miRNAs (12 known miRNAs and 7 novel miRNAs, Log2FoldChange ≥ 1, p ˂ 0.05) and 555 mRNAs (Log2FoldChange ≥ 1, p < 0.05) between the 2 groups. The differentially expressed genes were visualized using a volcano plot (Fig. 2A, B). Next, we attempted to identify putative miRNA–mRNA regulatory interactions to further investigate the role of miRNAs in diabetic testicular damage. Our strategy for identifying miRNA–mRNA regulatory relationships was based on 2 criteria: prediction of computational targets and negative regulation relationship. We used the Targetscan 7.2 database (http://www.targetscan.org/) to target gene prediction for miRNAs, and accordingly noted that 13,885 target mRNAs were predicted from 12 differentially expressed known miRNAs. We then applied a Venn diagram to obtain the intersection of the miRNA-predicted target genes and differentially expressed mRNAs according to the negative regulation (Fig. 2C). Finally, we selected 215 genes, and constructed a ceRNA regulatory network (Fig. 2D). To investigate the biological effects of miRNAs in the testes of diabetic rats, we performed KEGG pathway analysis on 215 selected target genes. Our results revealed that the PI3K-Akt signalling pathway (Alzahrani 2019), axon guidance, ECM-receptor interaction (Li et al. 2020; Yan et al. 2019), and MAPK signalling pathway (Yue and López 2020) were the top-scoring enrichments (Fig. 2E). Interestingly, most of these pathways are related to cell survival and apoptosis.

### Validation of miRNA expression in clinical specimens

**Fig. 3:**
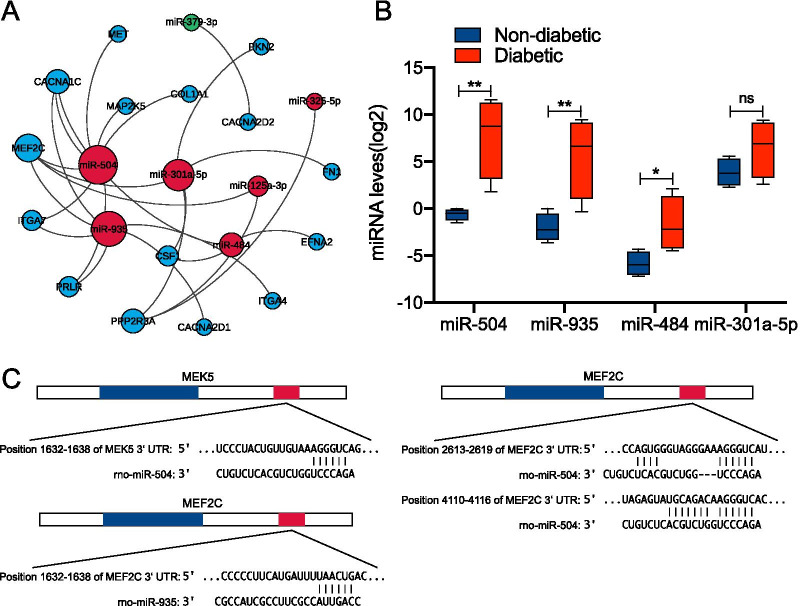
RT-qPCR analysis of differentially-expressed miRNAs. The miRNA–mRNA regulation network associated with the PI3K/AKT and MAPK pathways were constructed using the Gephi software (**A**). RT-qPCR analysis of differentially-expressed miRNAs (miR-504, miR-935, miR-484, miR-301-5p) in the serum of normal glucose tolerance subjects and type 2 diabetic patients (**B**). Data are presented as box plots, where all fold changes were calculated between medians. The y-axis indicates the expression level of miRNAs on a log2 scale. *p < 0.05, **p < 0.01, NS, not significant. The binding sites of miR-504 and miR-935 in the 3'-UTR of MEK5 and MEF2C mRNA were predicted using miRNA target prediction algorithms

We then aimed to gain further insight into the potential regulatory roles of miRNAs in the testicles of diabetic rats, whether in spermatogenic or somatic cells, and specifically their role in the survival and apoptosis of these cells. KEGG pathway analysis found that these miRNAs exerted their effect mainly through the PI3K/AKT and MAPK signalling pathways. We recreated the Ce regulatory network map of mRNAs and miRNAs that regulate them in the 2 classic survival and apoptotic pathways enriched in the PI3K/AKT and MAPK pathways through KEGG analysis. We found that the top-ranked 4 miRNAs that regulate a number of mRNAs were miR-504, miR-935, miR-484, and miR-301a-5P. We clinically collected the serum of young male (20–35 years old) patients with type 2 diabetes (the pathogenesis was all due to chronic consumption of high sugar diet and a family history of diabetes) to determine the expression of the aforementioned miRNAs. Compared with healthy volunteers (clinical information was shown in Additional file 1: Table S1), our results showed that the expression of miR-504, miR-935, and miR-484 in patients with type 2 diabetes was higher than that in healthy volunteers, and the difference between miR-504 and miR-935 was the most significant (Fig. 3B). This finding was consistent with the sequencing results. We further observed that the Ce regulatory network map identified MEF2C as one of the most miRNA-regulated mRNAs, with both miR-504 and miR-935 simultaneously targeting this gene. Interestingly, MEK5 (MAP2K5) in the MEK5-ERK5-MEF2C pathway that exists in MEF2C was also demonstrated to be regulated by miR-504. We hence assumed that miR-504 and miR-935 might co-regulate MEK5-ERK5-MEF2C via the classic survival pathway. To further clarify the regulatory relationship between miR-504, miR-935, MEK5, MEF2C, and their targets, we predicted the miRNA–mRNA seed-site interaction between them using the Targetscan 7.2 database. Our results revealed a putative binding site of miR-504 in the 3 untranslated region (3 UTR) of MEF2C mRNA. This indicated the presence of 2 putative binding sites of miR-504 in the 3 untranslated region (3 UTR) of MEF2C mRNA, one binding site with MEK5, and one binding site between miR-935 and the MEF2C3′ region (Fig. 3C).

### Glucose regulated the expression of miRNAs and biological functions of Leydig cells in a dose-dependent manner

**Fig. 4:**
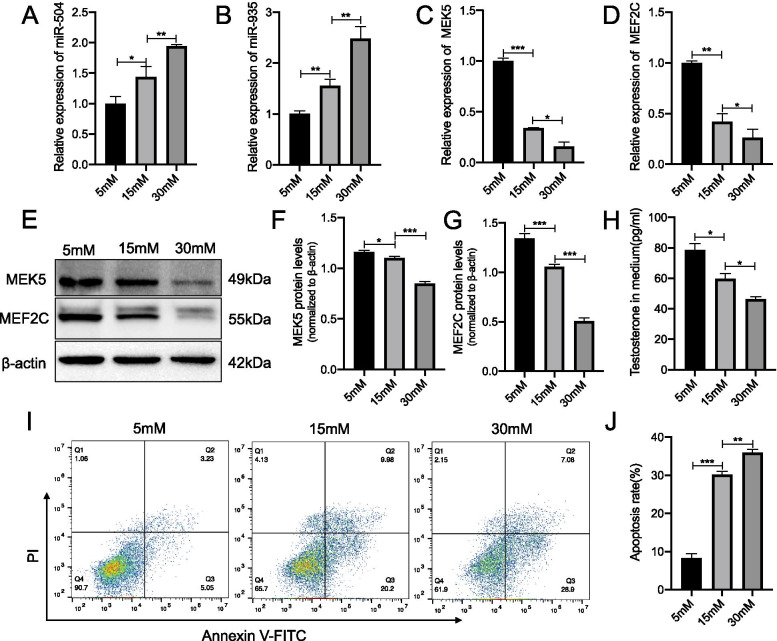
Effect of glucose concentration on miRNAs and apoptosis. Expression of miR-504 (**A**) and miR-935 (**B**) in R2C cells at 24 h after culturing in a glucose concentration gradient (basal glucose for R2C cell was 5 mM and stimulated concentrations were 15 mM and 30 mM). Data were normalised to U6 RNA, used as an internal control. Expression of MEK5 (**C**) and MEF2C (**D**) determined using RT-qPCR analysis. β-actin was used as an internal control. Representative immunoblotting (**E**) and cumulative quantification of the protein levels of MEK5 (**F**) and MEF2C (**G**) in R2C cells. Media were collected and assayed for concentration of testosterone using ELISA (**H**). Detection of apoptotic cells using FACS analysis with FITC-labelled annexin V and PI staining (**I**). Bar graphs represent the percentage of apoptotic cells in each group (**J**). *p < 0.05, **p < 0.01, ***p < 0.001. n = 3

To further explore the function of miR-504 and miR-935 in diabetic testicular cells, we used Leydig tumour R2C cells from rat testes to construct a high-glycaemic cell model. The reason for choosing Leydig cells was that diabetic patients exhibit decreased levels of androgen as a typical symptom (Kalyani and Dobs 2007). Although R2C cells are tumor cells, they have been used in multiple studies to establish models of cytotoxicity and androgen secretion (Deb and Bandiera 2011; Li et al. 2019a; Balbuena et al. 2013). Compared with R2C cells, the individual difference in Leydig cells isolated from diabetic rats (primary cells) is considered to be large which would seriously confound the results. Therefore, primary cells are not selected for subsequent experiments. Low levels of androgen are known to result in a series of reproductive system complications, such as reduced spermatogenesis and sexual desire, as well as erectile dysfunction (Minaz et al. 2019; Ding et al. 2015; Sajadi et al. 2019). Androgens are known to be primarily secreted by Leydig cells (Zirkin and Papadopoulos 2018). Therefore, the study of the role of miRNAs in the damage to testicular Leydig cells in diabetic individuals could provide good therapeutic targets and ideas for related treatments. We treated R2C cells with gradient concentrations of glucose (basal glucose for R2C cell was 5 mM and stimulated concentrations were 15 mM and 30 mM), and our results showed that the expression of miR-504 and miR-935 increased with increasing glucose concentrations (Fig. 4A, B), whereas the expression of the MEK5 and MEF2C downstream target genes was decreased with an increase in the concentration of glucose (Fig. 4C, D). We observed a similar trend in the changes of the MEK5 and MEF2C proteins (Fig. 4E–G). We then measured the testosterone content in the cell culture medium and the cell apoptosis rates. Our cell model simulated the microenvironment of Leydig cells in the testes of diabetic patients to some extent. We found that testosterone decreased with the increasing concentration of glucose, whereas the rate of apoptosis increased with the increasing concentration of glucose (Fig. 4I). These results indicated that glucose had a certain toxic effect on Leydig cells and could induce their apoptosis, in agreement with previous studies, which suggested that this toxic effect is regulated by the concentration of glucose. Besides, high levels of glucose were also found to induce an increase in miR-504 and miR-935 and the downregulation of MEK5 and MEF2C. This regulation was also demonstrated to be dependent on the concentration of sugars.

### miR-504 inhibited the proliferation and promoted the apoptosis of Leydig cells by targeting MEK5 and MEF2C

**Fig. 5:**
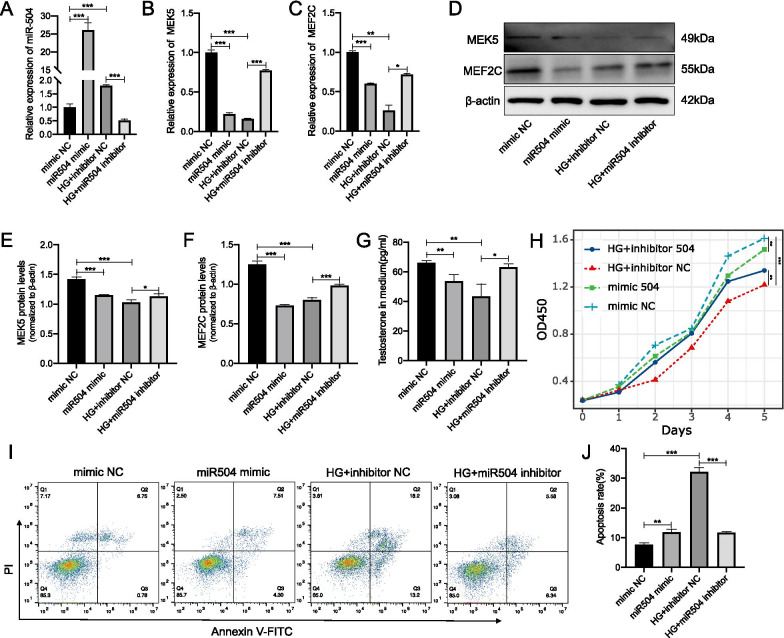
Modulation of proliferation and apoptosis of Leydig cells by mRNA targets of miR-504. Expression of miR-504 in miR-504 mimic-or miR-504 inhibitor-infected R2C cells at 24 h after culturing in normal or high glucose (HG). Data were normalised to U6 RNA, used as an internal control (**A**). Expression of MEK5 and MEF2C determined by RT-qPCR analysis. β-actin was used as an internal control (**B**, **C**). Representative immunoblotting (**D**) and cumulative quantification (**E**, **F**) of the protein levels of MEK5 and MEF2C in R2C cells transfected with miR-504 mimic, miR-504 inhibitor, mimic NC, or inhibitor NC. Media were collected and assayed for concentration of testosterone using ELISA (**G**). Cell proliferation was assayed using CCK8 (**H**). Detection of apoptotic cells by FACS analysis with FITC-labelled annexin V and PI staining (**I**). Bar graphs represent the percentage of apoptotic cells in each group (J). *p < 0.05, **p < 0.01, ***p < 0.001. n = 3

The aforementioned experiments demonstrated the effect of high glucose on the function of Leydig cells and their regulation by miR-504 and miR-935. However, whether miR-504 and miR-935 are involved in the damage of R2C cells under the effect of high glucose, and whether the downregulation of MEK5 and MEF2C is regulated by miR-504 and miR-935 remain unclear. Therefore, we conducted a series of studies on the role of miR-504 and miR-935 in R2C cells. We first used oligos to overexpress miR-504 in normal cultured R2C cells, and knock-down the expression of miR-504 on R2C cells cultured in a high-glucose environment (30 mM) (Fig. 5A). Next, we measured the expression of the 2 target genes, MEK5 and MEF2C, predicted by miR-504. Our results showed that the expression of MEK5 and MEF2C was significantly decreased, which was similar to the expression of MEK5 and MEF2C in a high-glucose environment. This decrease in the expression of MEK5 and MEF2C caused by high glucose was reversed when we knocked-down the expression of miR-504 in R2C cells cultured with high glucose (Fig. 5B, C), The above trends were consistent with the results of MEK5 and MEF2C protein assays (Fig. 5D–F). We then tested the cell phenotype of R2C. We first detected the secretion of testosterone in R2C cells. Our results showed that the overexpression of miR-504 could inhibit the secretion of cell testosterone, whereas knocking-down the expression of miR-504 could partially recover the high-glucose-induced weakened secretion of testosterone by R2C cells. Subsequently, we tested the proliferation and apoptosis of R2C cells and found that after overexpressing miR-504, the proliferation rate of R2C cells slowed–down, whereas apoptosis was increased. Knockdown of miR-504 reversed the decrease in the proliferation, whereas increased apoptosis caused by high levels of glucose (Fig. 5H–J).

### miR-935 inhibited the proliferation and promoted the apoptosis of Leydig cells by targeting MEF2C

**Fig. 6:**
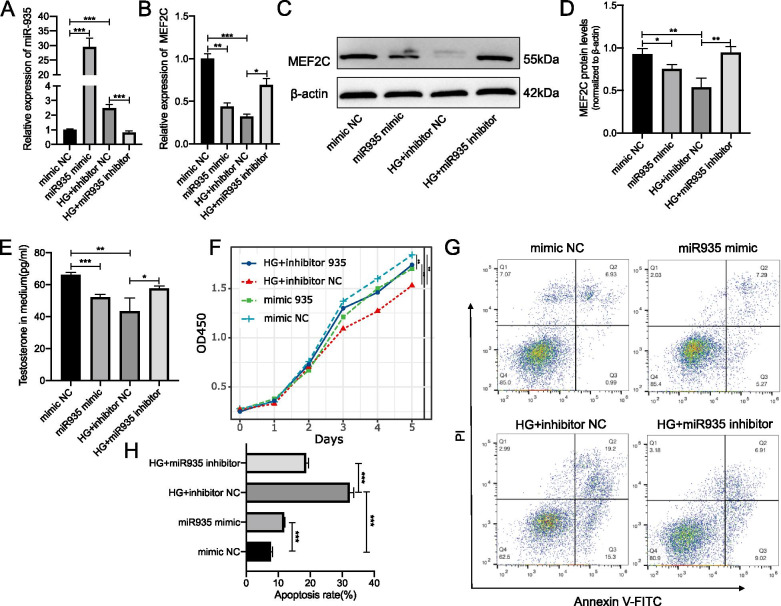
Modulation of proliferation and apoptosis of Leydig cells by mRNA targets of miR-935. Expression of miR-935 in miR-935 mimic-or miR-935 inhibitor-infected R2C cells at 24 h after culturing in normal or high glucose (HG). Data were normalised to U6 RNA used as an internal control (**A**). Expression of MEF2C determined by RT-qPCR analysis. β-actin was used as an internal control (**B**). Representative immunoblotting (**C**) and cumulative quantification (**D**) of the protein levels of MEF2C in R2C cells transfected with miR-935 mimic, miR-935 inhibitor, mimic NC, or inhibitor NC. Media were collected and assayed for concentration of testosterone using ELISA (**E**). Cell proliferation was assayed using CCK8 (**F**). Detection of apoptotic cells by FACS analysis with FITC-labelled annexin V and PI staining (**G**). Bar graphs represent the percentage of apoptotic cells in each group (H). *p < 0.05, **p < 0.01, ***p < 0.001. n = 3

Next, we performed a similar experiment using miR-935 in R2C cells. Our results showed that the expression of the MEF2C mRNA and protein was decreased (Fig. 6B–D) after the overexpression of miR-935 (Fig. 6A). We also found that the decreased secretion of testosterone (Fig. 6E) slowed-down the proliferation rate. This was similar to the biological changes observed in R2C cells in a high-glucose environment. However, we observed that when the expression of miR-935 was knocked-down in a high-sugar medium, the above phenotypes were reversed. The above 2 sets of experiments indicated that high glucose could induce the high expression of miR-504 and miR-935. The high expression of miR-504 and miR-935 might be negatively regulated by targeting MEK5 and MEF2C, thereby inducing cell apoptosis. As such, slowing-down the proliferation of R2C cells would result in the decreased secretion of testosterone.

## Discussion

**Fig. 7:**
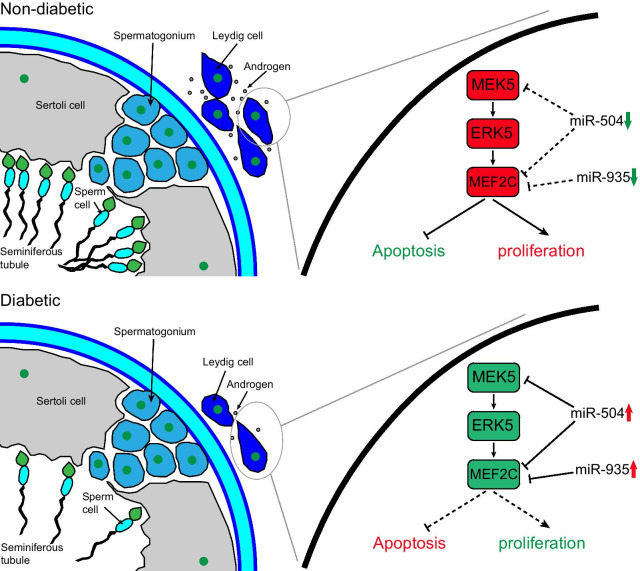
Schematic showing the molecular mechanisms of diabetes-induced testicular damage. Notes: In the diabetic state, the expression of miR-504 and miR-935 in Leydig cells increases, thereby inhibiting the MEK5/ERK5/MEF2C pathway, leading to increased interstitial cell apoptosis and inhibition of proliferation. This results in a reduced secretion of androgens, which in turn leads to a decrease in sperm production. Green indicates inhibition, whereas red indicates enhancement. Solid lines to indicate enhanced effects and dotted lines to indicate weakened stimulatory or inhibitory effects

The main findings of this study could be summarized in the following. The expression profile of testicular miRNAs differed significantly between diabetic and normal rats.The differentially expressed miRNAs and mRNAs formed together a miRNA–mRNA regulatory network, which was involved in multiple signal transduction pathways in diabetic testicular damage. The miR-504 and miR-935 collaborative inhibition of the classic survival pathway of MEK5-MEF2C in diabetic testis induced the apoptosis of Leydig cells and inhibited their cell proliferation, as shown in Fig. 7.

MicroRNAs (miRNAs) are small, non-coding RNA molecules that function by regulating the expression of target genes by either inducing the degradation or inhibiting the translation of mRNAs through imperfect base-pairing with the 3′-UTR of target mRNAs (Fabian and Sonenberg 2012). The miRNA pathways have been reported to be involved in diverse physiological and pathological processes, including self-renewal, proliferation, differentiation, and apoptosis. Key control factors and biomarkers have been demonstrated to serve as clinically specific biomarkers and therapeutic targets (Lu and Rothenberg 2018). Many studies have shown that miRNAs play an important role in various diabetes-induced organ damages (Chang and Wang 2019; Petrie et al. 2018; Vasu, et al. 2019). For instance, miR-301 and miR-449 have been shown to regulate the levels of DNA methyltransferase (DNMT) inhibitors and histone deacetylases (HDAC), thus participating in the development and progression of diabetic kidney disease (Sankrityayan et al. 2019). Likewise, miR-451a/ATF2 was reported to play a vital role in diabetic retinal pigment epithelial cell (RPE cell) disease by regulating the mitochondrial function (Shao et al. 2019). One study found that miR-30c exhibited a protective effect on diabetic cardiac metabolism via targeting PGC-1β (Yin et al. 2019). Moreover, miRNAs have also been reported to be involved in diabetic testicular damage. Recent studies revealed that miRNA-34a led to testicular cell apoptosis by targeting the sirtuin 1 (SIRT1) mRNA (Jiao et al. 2018), whereas nitrate could improve the testicular tissue architecture and function by increasing the level of miRNA-34b and reducing p53 mRNA, further increasing the fertility index (Keyhanmanesh et al. 2019). However, these studies did not describe the role and mechanism of miRNAs in diabetic testicular damage from a high-throughput perspective and none of them performed miRNA RNA-Seq for the identification of differentially expressed miRNAs between diabetic and non-diabetic testes. In this study, we discovered 12 known differentially expressed miRNAs. Through a series of bioinformatics analysis, we found that these miRNAs have a powerful effect in diabetic testicular damage. A number of intensive studies were carried out on miRNA-504 and miRNA-935. This was not only because their expression in the blood of diabetic patients was consistent with the sequencing results, but because they also play a common regulatory role in the classic survival pathway of MEK5-ERK5-MEF2C.

In particular, miR-504 has been widely studied in a number of different types of cancer and has been suggested to participate in the occurrence and development of a number of types of malignant tumours, such as nervous system tumours, haematological tumours, lung cancer, colon cancer, osteosarcoma, breast cancer, and liver cancer (Cai et al. 2017; Chen and Fu 2020; Cui et al. 2016; Gao 2019; Li et al. 2019b; Liu et al. 2019; Quan et al. 2018; Rong et al. 2018). In these studies, miR-504 was reported to mostly play a role in inhibiting tumour proliferation and promoting tumour apoptosis, consistent with the results of our current study. In addition, miR-504 was also shown to play a key role in mental illnesses, such as nicotine addiction (Huang and Li 2009). Importantly, miR-504 has also been involved in diabetic vascular smooth muscle damage, similar to the results of our study: Reddy et al. found that miR-504 was highly expressed in the vascular smooth muscle cells (VSMCs) of diabetic mice, resulting in the dysfunction of VSMCs by targeting Grb10 and Egr2 (Reddy et al. 2016). Additionally, miR-935 has been reported to play an important role in the differentiation of single-cell M2-like macrophage. Although a number of studies have indicated that miR-935 is involved in tumour metastasis, some reports have suggested that it could function to inhibit tumour proliferation. Despite the lack of any report on the involvement of miR-935 in diabetic organ damage, significant differences have been observed in its expression among obese people; this is especially interesting considering that obesity is an important cause of type 2 diabetes. In our study, we found that miR-504 and miR-935 were highly expressed in diabetic testes and upregulated in a sugar concentration-dependent manner in Leydig cells. Studies have demonstrated that miRNAs can regulate biological processes in 2 ways. A single miRNA might target multiple mRNAs and these mRNAs could jointly regulate the same molecule or cell process, or several miRNAs might coordinate to regulate the expression of a single mRNA that regulates a certain pathway or phenotype. We found that the regulation of miR-504 and miR-935 on diabetic testicular injury was synergistic and cumulative, reflecting the above 2 regulation pathways. More specifically, miR-504 was shown to simultaneously target and inhibit MEK5 and MEF2C, and as MEF2C is also known to be the target of miR-935, it was assumed that miR-504 and miR-935 jointly inhibit the MEK5-ERK5-MEF2C classical survival pathway. It is well known that the mitogen-activated protein kinase/extracellular signal-regulated kinase (MAPK/ERK) pathway is related to cell proliferation, differentiation, migration, senescence, and apoptosis (Safa et al. 2020). The MEK5 protein is the latest family member discovered in MAPK (Cristea et al. 2020; Hoang et al. 2017). It is known that ERK5 phosphorylates and activates the MEF2C downstream target transcription factor, which has transcriptional activity, thus promoting cell proliferation and inhibiting cell apoptosis (Herglotz et al. 2016; Honda 2015). The overexpression of miR-504 and miR-935 in diabetic testis Leydig cells was reported to synergistically inhibit the MEK5-ERK5-MEF2C survival pathway, regulate the proliferation and apoptosis of Leydig cells, and subsequently affect the secretion of androgens and sperm formation. Therefore, we have discovered a molecular regulatory miRNA–mRNA network involved in the pathological process of diabetic testicular damage, which is essential for the prevention of long-term testicular damage in diabetes.

However, our study had certain limitations. We did not carry out a luciferase reporter gene experiment to prove the direct regulatory relationship between a miRNA and its target gene and did not use miR-504 and miR-935 inhibitors for in vivo experiments. The main reason for this was that the main purpose of our research was to mine the key miRNAs in testicular injury based on RNA sequencing, and only verify their function. In future studies, we will further explore the role and regulatory mechanism of miR-504 and miR-935 in diabetic testes.

## Conclusion

We constructed a miRNA–mRNA molecular regulatory network using second-generation sequencing. Both miR-504 and miR-935 targeted the MEK5-ERK5-MEF2C survival pathway, inhibiting the proliferation, and promoting the apoptosis of testicular cells, resulting in a decrease in the secretion of androgens, which in turn led to a series of complications, such as reduced spermatogenesis and erectile dysfunction. Hence, miR-504 and miR-935 might be important targets for the future treatment of diabetic testicular damage. Accordingly, local inhibitors of these miRNAs could be developed to treat and prevent related symptoms in patients with diabetic testicular damage. Therefore, it is made apparent that the identification of key miRNAs that affect Leydig cells in a high-sugar environment is of great importance for the management of diabetes-induced reproductive-associated complications.

## Supplementary Information

**Additional file 1: Table 1.** Clinical information of healthy volunteers and type 2 diabetes patients

## Data Availability

The datasets generated and/or analysed during the current study are available in the GEO database (Accession code: GSE169131) repository. [https://www.ncbi.nlm.nih.gov/geo/query/acc.cgi?acc=GSE169131]. The datasets used and/or analysed during the current study are available from the corresponding author on reasonable request.
