# Supplementary material for: MicroRNA regulation of the proliferation and apoptosis of Leydig cells in diabetes
Source: Mol Med. 2021 Sep 8;27:104. doi: 10.1186/s10020-021-00370-8 (PMC8425090; doi:10.1186/s10020-021-00370-8)
Supplement: Supplementary file 1 — Additional file 1: Table 1. Clinical information of healthy volunteers and type 2 diabetes patients [file 10020_2021_370_MOESM1_ESM.docx]

|  | healthy volunteers  (n=20) | type 2 diabetes patients  (n=20) | p. value |
| --- | --- | --- | --- |
| Age | 27.60 | 26.25 | 0.3522 |
| BMI | 23.63 | 24.32 | 0.2240 |
| GLU (mmol/L) | 4.49 | 14.10 | ＜0.0001 |

Supplemental Table 1 Clinical information of healthy volunteers and type 2 diabetes patients

BMI: body mass index, GLU: fasting serum glucose
